# Image-guided in situ detection of bacterial biofilms in a human prosthetic knee infection model: a feasibility study for clinical diagnosis of prosthetic joint infections

**DOI:** 10.1007/s00259-020-04982-w

**Published:** 2020-09-08

**Authors:** Jorrit W. A. Schoenmakers, Marjolein Heuker, Marina López-Álvarez, Wouter B. Nagengast, Gooitzen M. van Dam, Jan Maarten van Dijl, Paul C. Jutte, Marleen van Oosten

**Affiliations:** 1 Department of Orthopaedics, University of Groningen, University Medical Center Groningen (UMCG), Groningen, The Netherlands; 2 Department of Medical Microbiology, University of Groningen, UMCG, Hanzeplein 1, PO Box 30001, 9700 RB Groningen, The Netherlands; 3 Department of Gastroenterology and Hepatology, University of Groningen, UMCG, Groningen, The Netherlands; 4 Department of Surgery, University of Groningen, UMCG, Groningen, The Netherlands

**Keywords:** Infection imaging, Prosthetic joint, Biofilm, Optical tracer, Fluorescence

## Abstract

**Purpose:**

Due to an increased human life expectancy, the need to replace arthritic or dysfunctional joints by prosthetics is higher than ever before. Prosthetic joints are unfortunately inherently susceptible to bacterial infection accompanied by biofilm formation. Accurate and rapid diagnosis is vital to increase therapeutic success. Yet, established diagnostic modalities cannot directly detect bacterial biofilms on prostheses. Therefore, the present study was aimed at investigating whether arthroscopic optical imaging can accurately detect bacterial biofilms on prosthetic joints.

**Methods:**

Here, we applied a conjugate of the antibiotic vancomycin and the near-infrared fluorophore IRDye800CW, in short vanco-800CW, in combination with arthroscopic optical imaging to target and visualize biofilms on infected prostheses.

**Results:**

We show in a human post-mortem prosthetic knee infection model that a staphylococcal biofilm is accurately detected in real time and distinguished from sterile sections in high resolution. In addition, we demonstrate that biofilms associated with the clinically most relevant bacterial species can be detected using vanco-800CW.

**Conclusion:**

The presented image-guided arthroscopic approach provides direct visual diagnostic information and facilitates immediate appropriate treatment selection.

**Electronic supplementary material:**

The online version of this article (10.1007/s00259-020-04982-w) contains supplementary material, which is available to authorized users.

## Introduction

Human life expectancy is presently higher than ever before. The need for biomaterials to replace arthritic or dysfunctional body parts by prosthetics has, therefore, never been greater [1]. Total joint replacement with a prosthesis is nowadays the most performed substitute, which usually contributes to an enhanced quality of life. Approximately 2% of the patients, however, experience device failure in the form of a bacterial infection of the prosthesis and adjacent tissue [2]. These prosthetic joint infections (PJIs) are dreaded complications. They are difficult to diagnose, can manifest at any time after arthroplasty, and usually require multiple surgeries and a prolonged course of antibiotic treatment [3–5]. The economic impact of PJIs is accordingly significant with yearly estimated treatment costs exceeding $1.5 billion in the USA alone [2].

Implanted biomaterials are highly susceptible to infection due to biofilm formation on the prosthetic surface. A bacterial biofilm is a sessile community of bacteria surrounded by a matrix of extracellular polymeric substances (EPS) [6]. The matrix, which is produced by the bacteria, acts as a structural network. It captures and disseminates nutrients and functions as a barrier for the host immune system and antibiotics. These properties make the eradication of bacterial biofilms challenging. Moreover, the bacterial tolerance to therapeutic interventions increases as the biofilm matures [7]. This calls for innovative diagnostic tools that allow their early detection.

Established diagnostic modalities for PJIs, such as blood tests, analysis of synovial fluid, culturing, biopsies, and imaging all have their pros and cons in detecting infection, but no test is 100% accurate for PJI [8–10]. As a result, the diagnosis often remains unclear, whereas the proper therapeutic approach is uncertain. One factor that consistently plays a role in biomaterial-associated infection, however, is bacterial biofilm formation. Up to date, there are no diagnostic procedures capable of in vivo detection of bacterial biofilm on a (total) joint prosthesis.

**Fig. 1:**
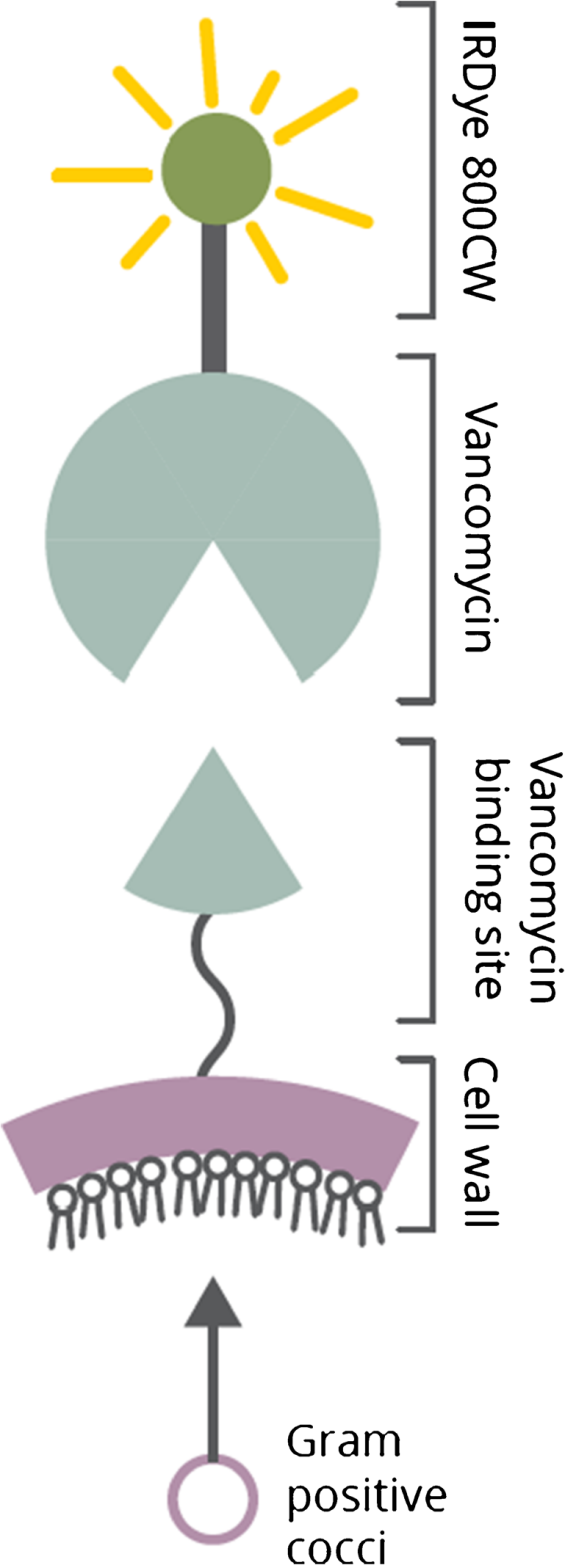
Schematic representation of vanco-800CW and its binding to the bacterial cell wall. The fluorescent tracer vanco-800CW is a conjugate of the antibiotic vancomycin and the near-infrared fluorophore IRDye800CW. It binds to the D-Ala-d-Ala moieties of *N*-acetylmuramic acid and *N*-acetylglucosamine peptides in the cell wall of Gram-positive bacteria [12]

Targeted fluorescence (i.e., optical) imaging (TFLI) has great potential for the in vivo detection of bacterial infections [11]. It relies on the administration of a tracer that consists of an exogenous fluorophore conjugated to a bacteria-targeting molecular probe. After administration of the tracer, a sensitive optical camera readily detects the fluorescence emission at the infection site and converts this into an image on a screen. This allows for high-resolution, low-cost imaging in real time. A promising tracer is vanco-800CW, which is a conjugate of the antibiotic vancomycin and the near-infrared (NIR) fluorophore IRDye800CW (Fig. 1) [12]. Vanco-800CW is particularly suitable for the detection of PJIs as vancomycin targets the cell wall of Gram-positive (GP) bacteria. Bacteria belonging to this class are the most frequently encountered causative agents of biomaterial-associated infections [8]. Previously, our team has demonstrated that this tracer can be successfully applied to detect a range of planktonic GP bacteria, to detect staphylococcal infections in mice, and to image an infected implant through several millimeters of human skin [12].

The present proof-of-principle study was aimed at evaluating the possible use of vanco-800CW for detection of PJIs. A human post-mortem prosthetic knee infection model was used to assess the clinical feasibility of detecting bacterial biofilms through TFLI. An arthroscopic approach was chosen for imaging the bacterial biofilms directly intra-articularly on the implanted prosthesis. In addition to the arthroscopy, an in vitro experiment was performed to verify that vanco-800CW is a suitable tracer for the detection and imaging of a representative panel of clinical PJI-causing bacterial isolates.

## Materials and methods

### Human post-mortem experiments

The post-mortem experiments were performed in duplicate, using the legs of two human cadavers, where each leg was used only once. Both individuals had provided written informed consent for the post-mortem use of their bodies for scientific research. All post-mortem experiments were conducted in accordance with the applicable law (“Wet op de Lijkbezorging,” Art 18, lid 1 and 19, BWBR0005009) and institutional guidelines of the UMCG.

#### Biofilm formation on the lateral part of a knee prosthesis

Before implantation in human cadaver knee joints, cobalt-chrome knee prostheses (size 5 triathlon femoral component; Stryker®, USA) were coated with a biofilm of *Staphylococcus epidermidis*. To this end, the *S. epidermidis* American Type Culture Collection (ATCC) strain 35984 was cultured overnight in 10 mL Tryptic Soy Broth (TSB) at 37 °C. One milliliter of the *S. epidermidis* culture with an optical density at 600 nm (OD_600_) of 0.1 was transferred to a sterile container with 199 mL TSB, 5% glucose, and 4% sodium chloride (NaCl). Next, a knee prosthesis was incubated in the suspension with the lateral segment submerged. Accordingly, in this standing culture system, biofilm formation would only occur at the submerged lateral prosthetic region. This allowed for distinction during the arthroscopy between parts with biofilm (lateral side) or without biofilm (medial side). The prosthesis was biofilm-coated in 4 days at 37 °C. During this period, medium was manually refreshed once per day by removing 199 mL of suspension and replacing it by 199 mL of fresh TSB with 5% glucose and 4% NaCl.

Formation of a biofilm matrix of EPS on the knee prosthesis was verified by growing the biofilms in the presence of 0.2 mg/mL or 20 mg/mL Congo Red stain (Sigma-Aldrich, Germany) for 4 days at 37 °C as described above [13]. Subsequently, the prosthesis was washed twice with phosphate-buffered saline (PBS) and incubated for 30 min with Congo Red (0.004 mg/mL). Lastly, the prosthesis was washed twice with PBS to remove any unbound stain. Images of the stained biofilm on the prosthesis were recorded with a photographic camera.

#### Total knee arthroplasty

**Fig. 2:**
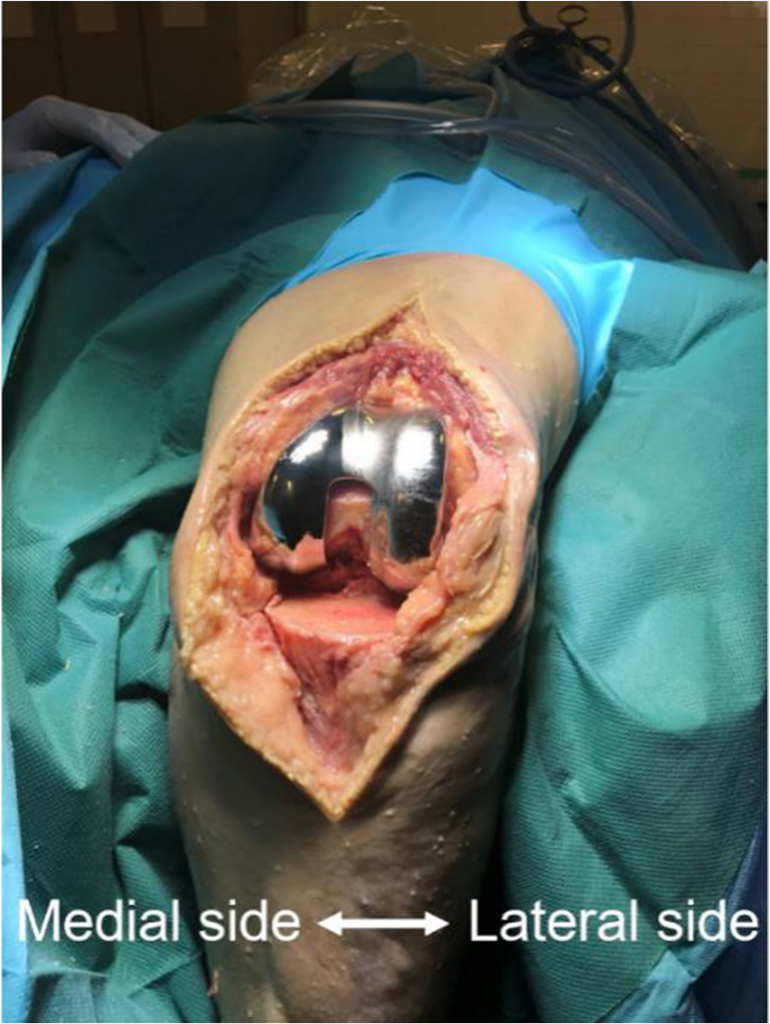
Image from the anterior side of the biofilm-coated prosthesis after implantation in the left knee of a human cadaver. The lateral part of the prosthesis was biofilm-coated with *S. epidermidis*, whereas the medial part was essentially sterile

The human cadaver knee joint was disinfected and draped. A longitudinal midline incision anterior of the knee joint was made, the joint was opened in a medial parapatellar fashion to gain access to the knee, and the patella was everted laterally. Subsequently, the knee joint was placed in 90° of flexion. The distal femoral bone cuts were performed using an oscillating saw to allow accurate placement of the femoral component of the prosthesis. A biofilm-coated prosthesis (as described above) was implanted on the distal femur (Fig. 2). Suturing of the soft tissues was performed in a double layer technique to allow for watertight closure (Ethilon 3-0, Ethicon Somerville, NJ, USA).

#### Imaging procedures and incubation with vanco-800CW

The knee was placed in 90° of flexion. An intra-articular portal was made by a small incision of 1 cm lateral to the lateral border of the patella tendon in the soft spot just distal to the pole of the patella. An arthroscope (Arthrex, Naples, FL, USA) connected to a saline irrigation pressure pump was placed inside the knee joint cavity. Physiological saline solution (“saline”) was pumped into the joint for distention. The prosthesis was then imaged with a white-light and NIR light camera (SurgVision Explorer Custom, Netherlands) with a fluorescence fiber attached to it (Schoelly Fiberoptic GmbH, Germany) as a control prior to the injection of vanco-800CW. The saline was sucked out of the knee with the irrigation system after this first round of imaging.

**Fig. 3:**
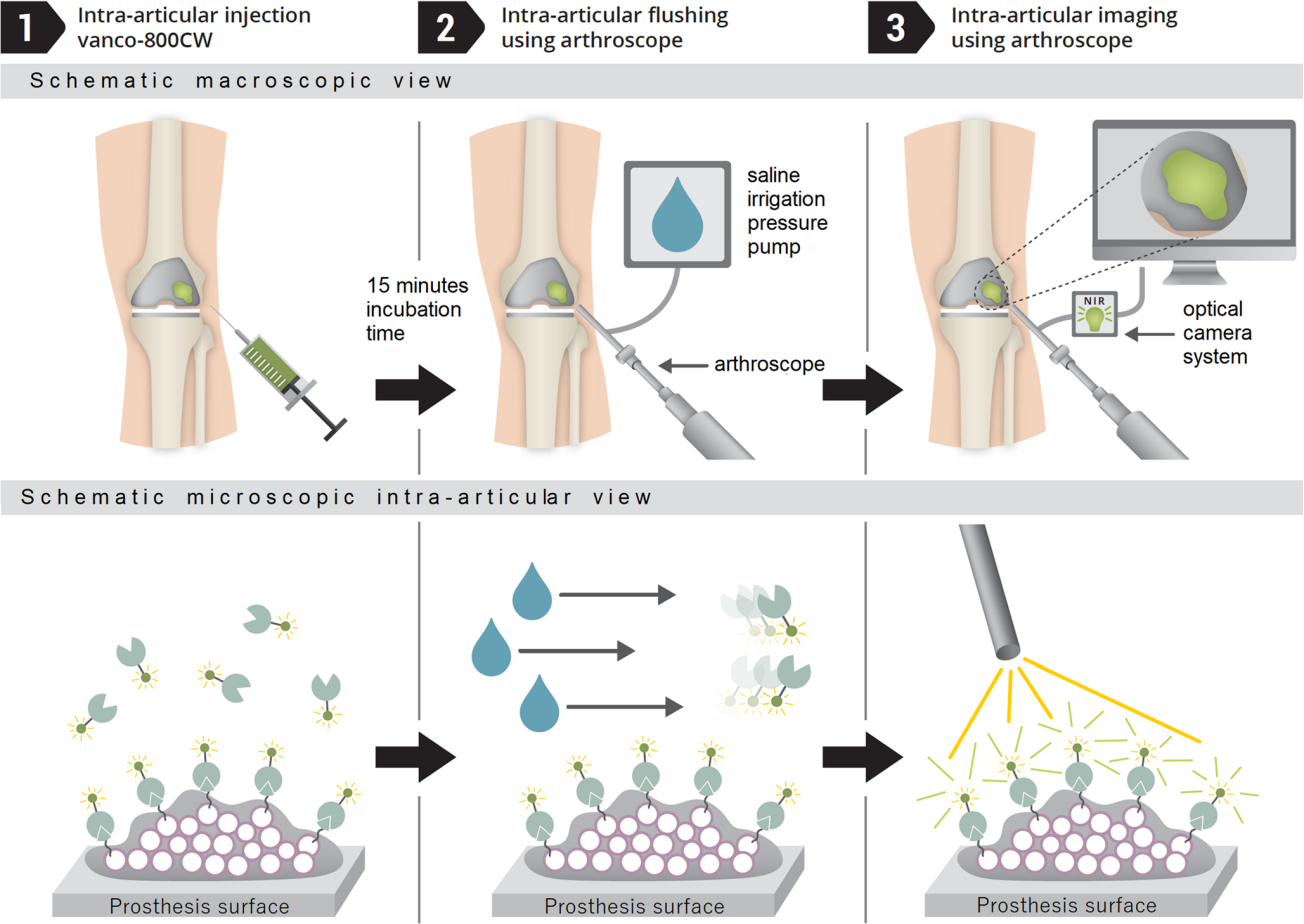
Schematic overview of arthroscopic biofilm imaging using vanco-800CW. (1) Vanco-800CW is injected intra-articularly, where it binds to the cell wall of biofilm-resident Gram-positive bacteria. (2) By arthroscopically flushing the joint with physiological saline solution, unbound vanco-800CW is removed. As a consequence, this tracer is solely retained in areas where it is bound to Gram-positive bacteria in the biofilm. (3) The joint is arthroscopically imaged in the near-infrared range. Fluorescence of vanco-800CW bound to bacteria in the biofilm is visualized on a screen. A video showing the procedure is provided as supplemental Movie 1

After imaging, the knee was placed in full extension and 20 mL of vanco-800CW dissolved in saline (0.07 nmol/mL, LI-COR Biosciences, NE, USA) was injected into the knee joint cavity (Fig. 3-1). The joint was then manually flexed and extended five times to ensure an even dispersion of the tracer. After a 15-min incubation period in extension, the knee joint cavity was thoroughly flushed with the arthroscope irrigation system with 2 L saline to remove all unbound tracer as well as any planktonic bacteria that could potentially be present (Fig. 3-2). Thereafter, the imaging procedure was repeated (Fig. 3-3).

### TFLI of biofilms of clinical bacterial isolates using vanco-800CW

A representative panel of commonly encountered PJI-causing bacterial pathogens, both GP and Gram-negative (GN), was collected from the diagnostic laboratory of the UMCG. All clinical isolates were retrieved from sonicated infected biomaterials from patients. Ethical approval for the collection of patient samples was obtained from the medical ethical committee at the UMCG (METC 2017/526). Biofilms of these strains, plus a biofilm of the *S. epidermidis* ATCC 35984 strain (used in the post-mortem experiments), were formed on cobalt-chrome discs (diameter 2.5 cm) in 4 days following the same inoculation and standing culture protocol as described for the post-mortem experiments above. These biomaterials, plus one sterile control containing no biofilm, were incubated with vanco-800CW (0.07 nmol/mL) and washed twice with PBS. White-light and NIR images were recorded using a fluorescence camera (SurgVision Explorer Air, Netherlands). The formation of EPS in the *S. epidermidis* biofilm was assessed by staining with Congo Red as described above [13]. The stained and imaged biomaterials were sonicated after which serial dilutions of the sonication-fluid were made. Samples were plated on blood agar plates (5% sheep blood, Mediaproducts B.V., the Netherlands), and numbers of colony-forming units (CFUs) were determined. The experiment was performed in duplicate.

### Fluorescence microscopy of *S. epidermidis* biofilms

*S. epidermidis* ATCC 38984 was grown overnight at 37 °C in TSB using a shaking incubator. Subsequently, the bacteria were diluted in TSB supplemented with 5% glucose and 4% NaCl to a final OD600 of 0.1 and incubated in a 12-well microtiter plate containing 18 mm chemically resistant borosilicate glass coverslips for microscopy (Paul Marienfeld GmbH, Germany). After 48 h of incubation, the coverslips were incubated for 15 min with vancomycin at 0.5, 1, 2, 4, or 8 mg/L final concentration or without vancomycin. Subsequently, the coverslips were washed once with PBS, incubated with 0.07 nmol/mL of vancomycin-BODIPY™ FL (vanco-BODIPY) for 15 min (Thermo Fisher Scientific, USA), washed once with PBS to remove any unbound tracer, and fixed with 4% paraformaldehyde. Finally, the coverslips were mounted on microscopy slides. Image acquisition was performed with a Leica TCS SP8X microscope.

### Data analysis

Fluorescence images of the post-mortem experiments and the biofilm experiments with clinical bacterial isolates were analyzed using the ImageJ software package (National Institutes of Health, MD, USA). The detection limit for the fluorescence signal was set at the lowest value at which biofilm signals were visually easily discernible from the negative control signal at biofilm-free sites. For the fluorescence assay, regions of interest (ROIs) were drawn around fluorescent biofilm–coated regions on the biomaterials after which the software quantified the fluorescence signal. The background fluorescence was quantified by drawing a ROI off-target in a background area of the same image. To determine the target-to-background (T/B) ratio, ROIs were divided by the background fluorescence. Graphs were plotted using GraphPad Prism 8.1.1 (GraphPad Software, CA, USA). The fluorescence microscopy images recorded to assess competition of the antibiotic vancomycin with vanco-BODIPY for binding to *S. epidermidis* ATCC 38984 were processed using Imaris 9.5.0 software (Oxford Instruments, UK).

## Results

### Human post-mortem experiment

**Fig. 4:**
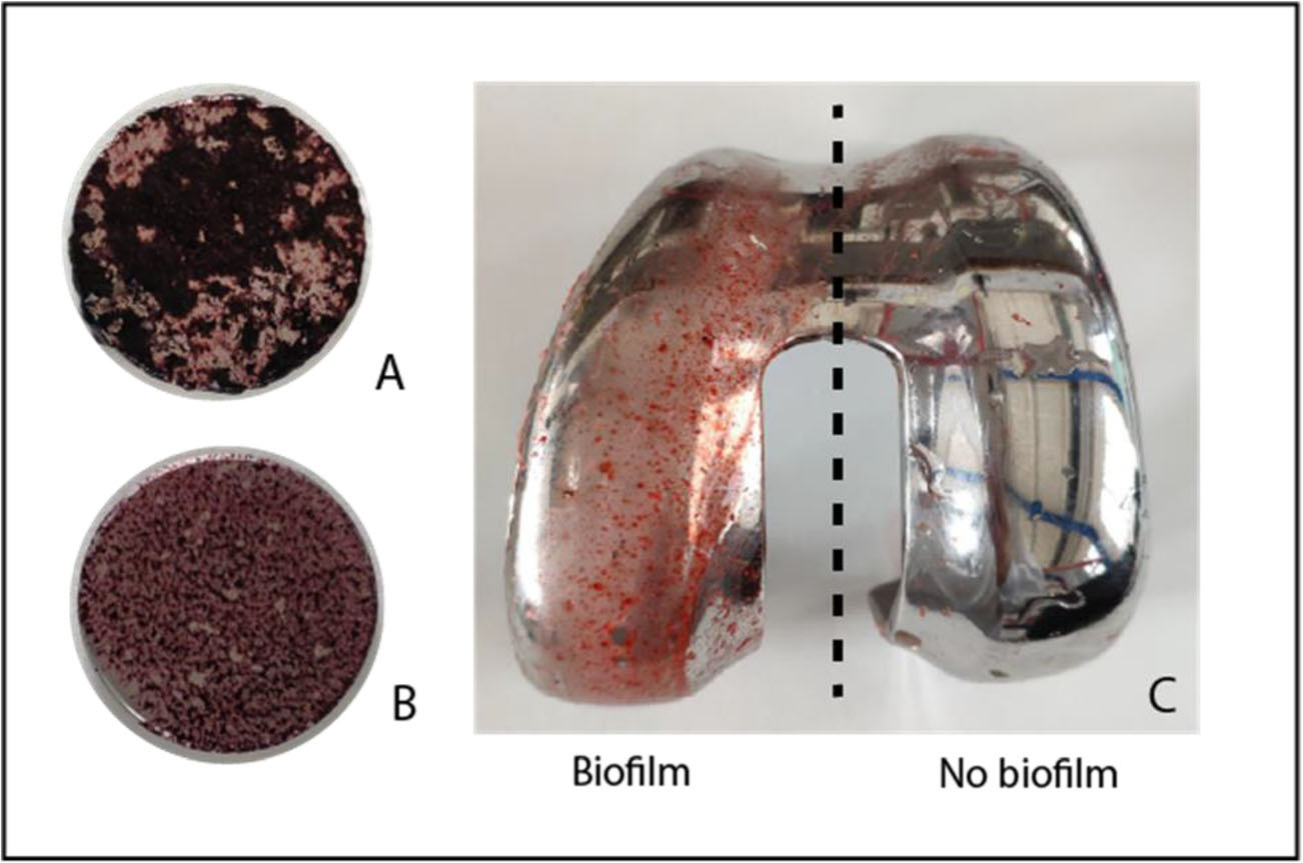
*S. epidermidis* biofilms stained with Congo Red. *S. epidermidis* biofilms were grown in 4 days on cobalt-chrome discs in the presence of 20 mg/mL (A) or 0.2 mg/mL (B) Congo Red. The *S. epidermidis* biofilm on the cobalt-chrome knee prosthesis, formed after 4 days of culturing, was stained for 30 min with 0.004 mg/mL Congo Red (C). Red staining of the biofilms marks the presence of extracellular polymeric substances (EPS)

To assess the formation of genuine *S. epidermidis* biofilms on cobalt-chrome biomaterials, we investigated the presence of EPS by staining the bacteria that had adhered to discs and knee prostheses with Congo Red [13]. This showed that after 4 days of culturing, *S. epidermidis* had indeed formed EPS-containing biofilms on these biomaterials (Fig. 4).

**Fig. 5:**
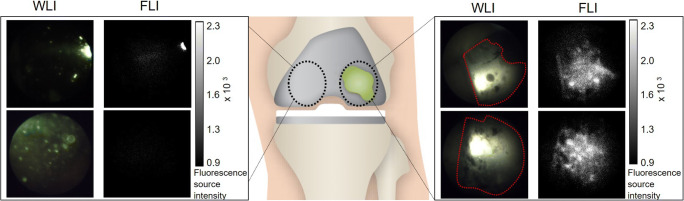
Fluorescence arthroscopic imaging of bacterial biofilms in a human post-mortem knee implant model. The green stain represents a *S. epidermidis* biofilm, applied in vitro prior to arthroplasty. Images were obtained after 15 min incubation with vanco-800CW and subsequent washing with saline. (A) White-light image (WLI) of the uncoated biofilm-free part of the prosthesis. (B) Corresponding near-infrared fluorescence image (FLI) of the uncoated part of the prosthesis. The bar on the right marks the corresponding fluorescence signal intensity. The bright spot in the upper image is caused by white-light reflection on the implant. (C) WLI of the biofilm-coated part of the prosthesis. The biofilm-coated areas are highlighted by the red dotted lines. (D) Corresponding near-infrared FLI of the biofilm-coated part of the prosthesis. The bar on the right marks the corresponding fluorescence signal intensity. Settings for fluorescence measurements: exposure 200 ms and gain 300

**Fig. 6:**
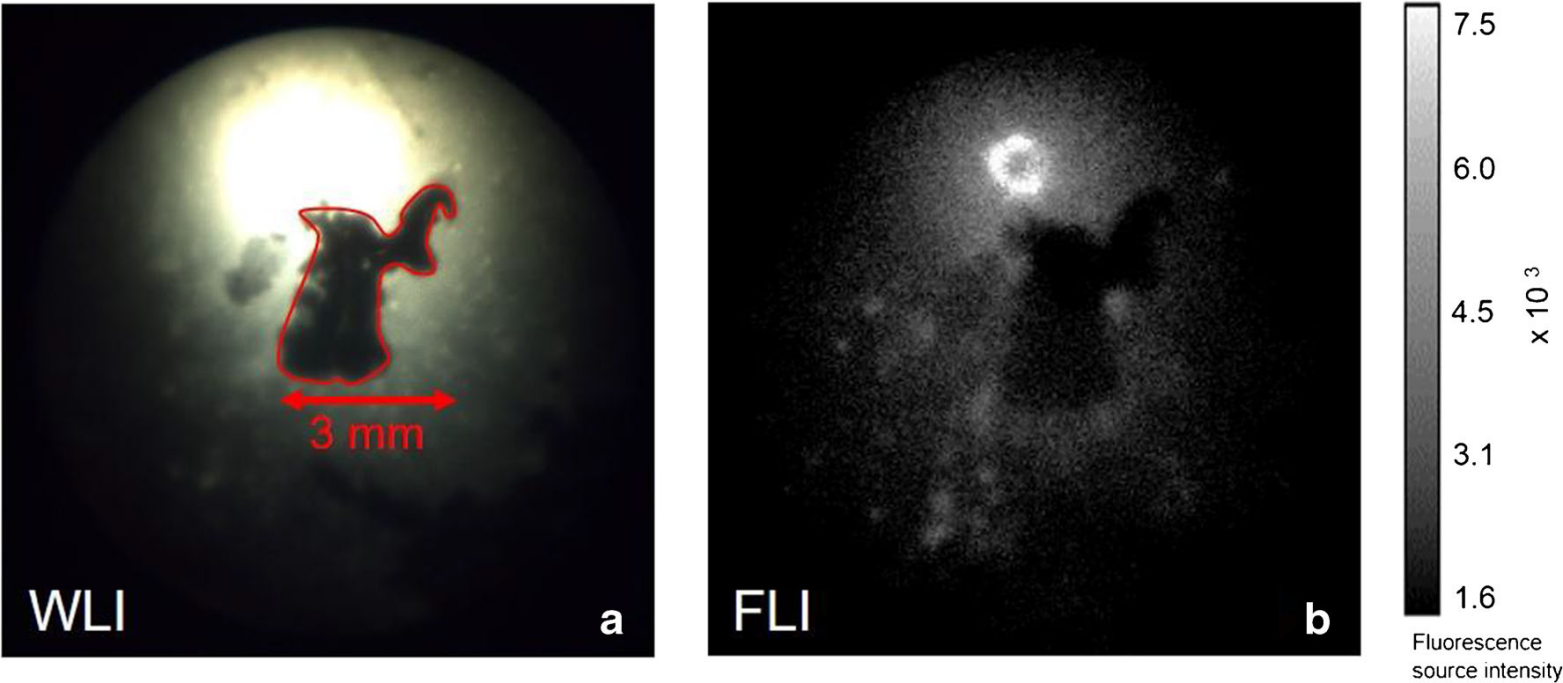
Close-up arthroscopic image of a *S. epidermidis* biofilm in a human post-mortem prosthetic knee model. Images were obtained after 15 min incubation with vanco-800CW and subsequent washing with saline (A) White-light image (WLI) of the biofilm. There is a 3-mm wide interruption in the biofilm-coated part where biofilm is missing, which is highlighted by the red line. The bright spot above the highlighted area is caused by white-light reflection on the implant. (B) Near-infrared fluorescence image (FLI) of the biofilm. The bar on the right marks the corresponding fluorescence signal intensity. Note the absence of fluorescence signal at the biofilm-free part of the prosthesis, which correlates with the zone highlighted by the red line in panel A. Settings for fluorescence measurements: exposure 800 ms and gain 300

The clinical feasibility of detecting bacterial biofilms through TFLI was evaluated by total knee arthroplasty with prostheses coated with (unstained) *S. epidermidis* biofilms on the lateral part and subsequent arthroscopy. Arthroscopic images captured by the white-light camera revealed the spatial distribution of the *S. epidermidis* biofilm on the lateral part of the prosthesis (Fig. 5-C, red dotted line). At the uncoated medial part of the prosthesis, no biofilm was visible (Fig. 5-A). Prior to the administration of vanco-800CW, no NIR fluorescence signal was detectable at both the lateral and the medial side of the prosthesis compared with the background. Upon incubation with vanco-800CW and subsequent washing with saline, the recorded NIR images revealed a strong fluorescence signal emitted from the biofilm-coated lateral part of the prosthesis (Fig. 5-D), whereas the uncoated medial part showed no fluorescence signal, comparable to the background (Fig. 5-B). Co-localization revealed a high degree of overlap between the white-light camera image and the fluorescence image (Fig. 5-C, D). Close-up images of the biofilms captured with the white-light camera and the NIR fluorescence camera were subsequently compared. Minor interruptions in the biofilm of only several millimeters wide (Fig. 6-A) were detectable with the white-light camera. These shapes could easily be recognized by the NIR fluorescence camera (Fig. 6-B), providing proof-of-principle that vanco-800CW and arthroscopic optical imaging can be applied for high-resolution visualization of bacterial biofilms on infected prostheses.

### TFLI to detect biofilms of clinical bacterial isolates using vanco-800CW

**Fig. 7:**
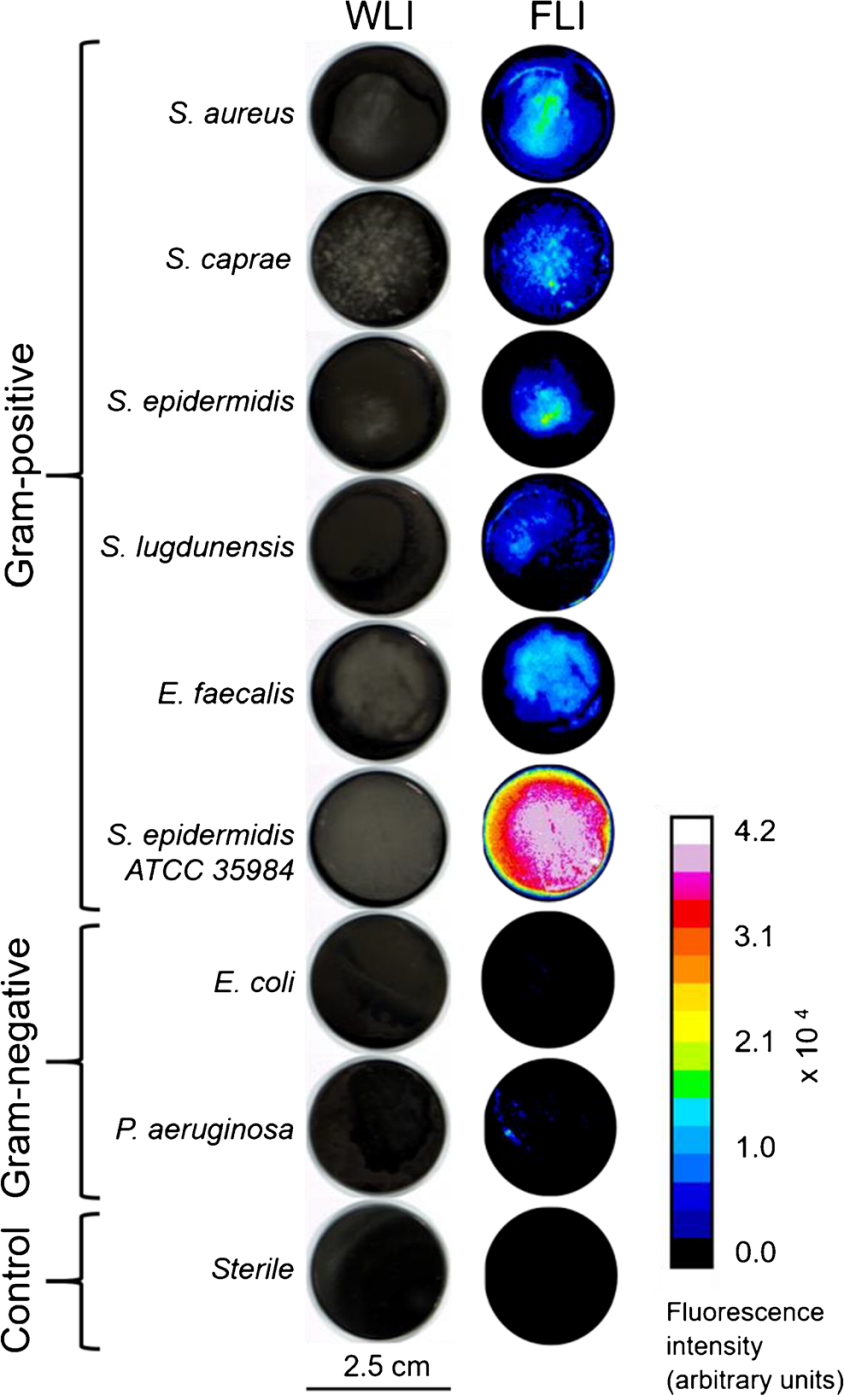
White-light images (WLI) and fluorescence images (FLI) of bacterial biofilms on cobalt-chrome biomaterials after treatment with vanco-800CW. The bacterial isolates were derived by sonication of infected prostheses with the exception of the *S. epidermidis* ATCC 35984 strain, which was also used in the post-mortem experiments. Images were obtained after 15 min incubation with vanco-800CW and subsequent washing with PBS. Of note, the *S. epidermidis* ATCC 35984 strain is known for its ability to rapidly form thick biofilms [14]*.* Consequently, the biofilms formed by the ATCC 35984 strain were thicker and bound more vanco-800CW than those formed by the clinical isolates. The bar on the right marks the correspondence of color in the FLI to fluorescence signal intensity. Settings for fluorescence measurements: exposure 25 ms and gain 300

**Fig. 8:**
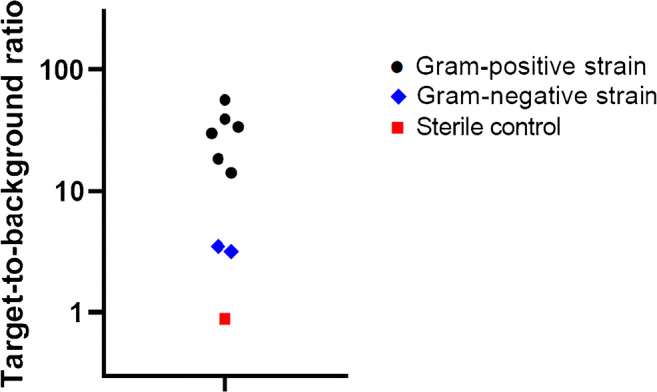
Average target-to-background (T/B) ratios for bacterial biofilms stained with vanco-800CW. Bacterial strains used to grow duplicate biofilms on cobalt-chrome biomaterials were derived by sonication of infected prostheses as described for Fig. 7. Upon staining of the biofilms with vanco-800CW, the average T/B ratios were determined for each strain. The Gram-positive bacterial strains included clinical isolates of *S. aureus*, *S. epidermidis*, *S. lugdunensis*, *S. caprae*, and *E. faecalis*, as well as the *S. epidermidis* ATCC 35984 strain. The Gram-negative bacterial strains included clinical *E. coli* and *P. aeruginosa* isolates. Fluorescence signals were quantified with ImageJ software

A representative panel of 14 clinical bacterial isolates was collected and used to grow in vitro biofilms on cobalt-chrome discs. The panel consisted of *Staphylococcus aureus* (2 isolates, GP), *S. epidermidis* (2 isolates, GP), *Staphylococcus lugdunensis* (2 isolates, GP), *Staphylococcus caprae* (2 isolates, GP), *Enterococcus faecalis* (2 isolates, GP), *Escherichia coli* (2 isolates, GN), and *Pseudomonas aeruginosa* (2 isolates, GN). In addition, biofilms of the *S. epidermidis* ATCC 35984 strain were grown on duplicate discs for control. Results of the subsequent biofilm imaging are shown in Fig. 7 (note that per strain only one image is shown). The biofilms of all GP bacterial isolates emitted a strong fluorescence signal (well above 1.0 × 10^4^ fluorescence units). In contrast, the GN bacterial isolates emitted a substantially lower fluorescence signal (0.5–1.0 × 10^3^ fluorescence units), as was to be expected because vancomycin particularly targets GP bacteria. The fluorescence signal detectable for the sterile controls was comparable to the background (< 0.5 × 10^3^ fluorescence units). After sonication and plating of the sonicates, the CFUs measured for the GP isolates (median 1.9 × 10^9^) were comparable to the CFUs measured for the GN strains (median 3.5 × 10^8^; see Supplementary Table 1 for the CFU counts and fluorescence measurements per ROI per species). Calculated average T/B ratios per species are shown in Fig. 8. All the GP strains showed a substantially higher T/B ratio (range 14.2–56.5, median 31.8) than the GN strains (range 3.2–3.5, median 3.4). The T/B ratios for the sterile biomaterials were 1, meaning that the sterile control presented the same fluorescence intensity as the unstained background.

### Competitive inhibition of *S. epidermidis* biofilm staining with fluorescent vancomycin by unlabelled vancomycin

**Fig. 9:**
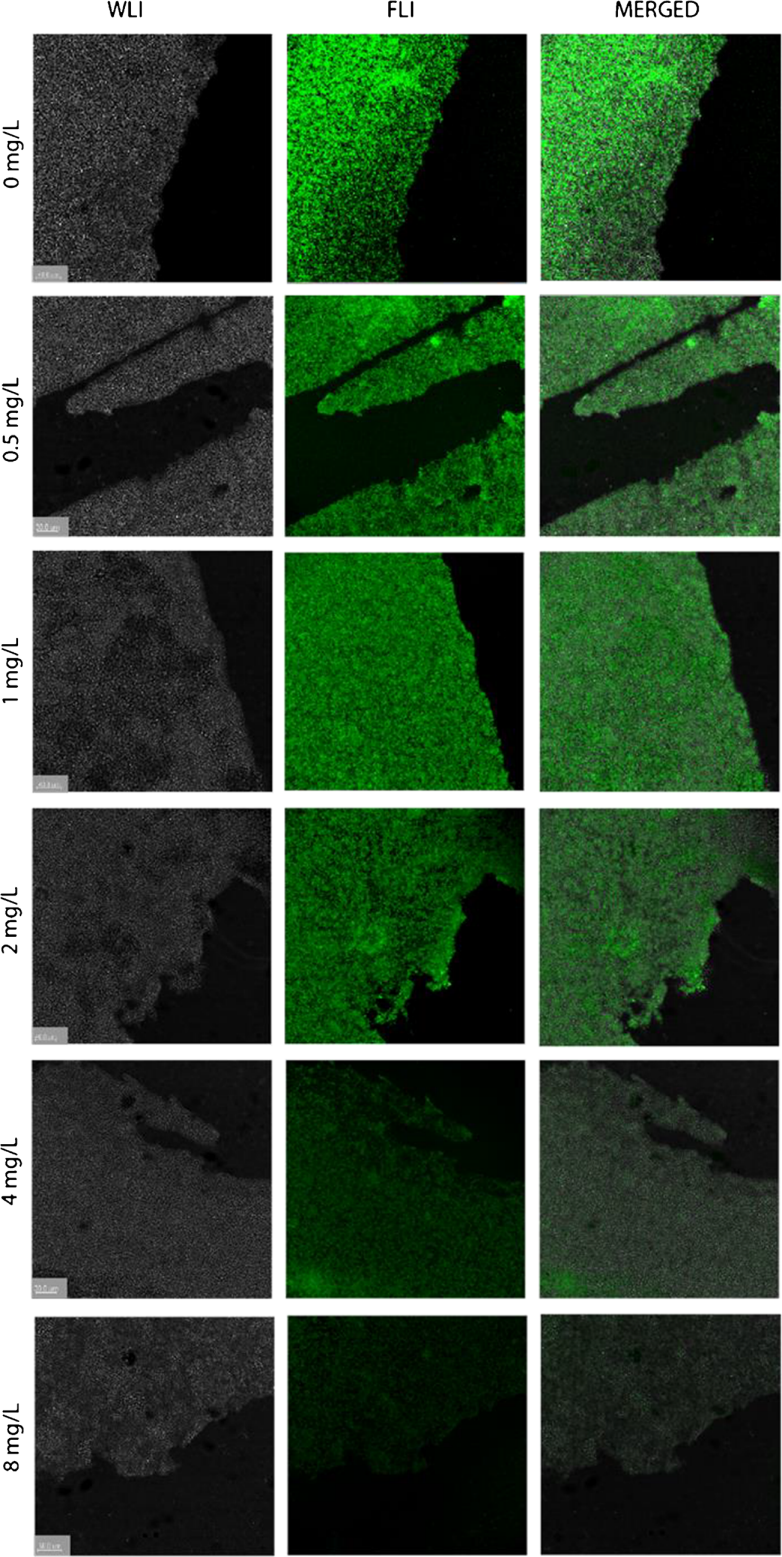
Competitive inhibition of *S. epidermidis* biofilm staining with fluorescent vancomycin by increasing concentrations of unlabelled vancomycin. *S. epidermidis* biofilms were grown on microscopy coverslips. The biofilms were incubated for 15 min with different concentrations of unlabelled vancomycin (up to 8 mg/L; shown on the left), or without vancomycin. After washing with PBS, the biofilms were stained with vanco-BODIPY for 15 min. White-light (WLI) and fluorescence images (FLI) were recorded with a Leica TCS SP8X microscope (magnification × 40)

To ascertain that the observed staining of GP bacterial biofilms by the fluorescently labelled vancomycin was vancomycin-specific, we performed a blocking experiment with unlabelled vancomycin. To this end, *S. epidermidis* biofilms grown on microscopy coverslips were treated with unlabelled vancomycin prior to the incubation with fluorescently labelled vancomycin and fluorescence microscopy. In this case, we used vanco-BODIPY instead of vanco-800CW, as vanco-BODIPY was more suitable for high-resolution fluorescence microscopy of biofilms in our setup. The results of the fluorescence microscopy analyses are shown in Fig. 9. Indeed, pre-treatment of the biofilms with vancomycin at concentrations higher than 2 mg/L resulted in a gradual decrease of biofilm staining by vanco-BODIPY. This shows that the observed labelling with fluorescent vancomycin derivatives is vancomycin-specific.

## Discussion

In this proof-of-principle study, we show that arthroscopic detection and real-time imaging of bacterial biofilms on a human knee prosthesis are feasible with vanco-800CW. In addition, we demonstrate that this tracer is highly effective in tracking biofilms formed by representative clinical GP bacterial isolates. As current diagnostic modalities for the detection of PJIs often yield inconclusive results, successful treatment can be hampered and lead to an increased patient- and healthcare burden. Therefore, the development of fast and accurate novel bacteria-targeting diagnostic modalities is desirable. The potential advantages of arthroscopic TFLI in comparison with present diagnostic modalities for PJIs are considerable. The presently shown ability of TFLI to detect and image bacterial biofilms in situ on a prosthesis, both in high-resolution and in real time, are aspects that mark this technology’s exceptional potential for clinical implementation.

Especially in oncological research, pioneering clinical studies have demonstrated the benefits of TFLI-guided detection of cancer, surveillance biopsies, and surgical resection [15, 16]. To date, infection imaging with TFLI has never been done in patients but has proven extremely promising in vitro and in vivo [11]. Due to recent (technical) advances concerning fluorophores, targeting molecules, and optical camera systems, the first-in-man studies exploiting TFLI in infection imaging are expected within the next few years according to Mills et al. [17]. Moreover, fluorescently labelled antimicrobial peptides and antibiotics, such as vanco-800CW, are likely to be the first tracers used in clinical practice. Before clinical introduction, vanco-800CW awaits toxicity testing and good manufacturing practices to ensure its safety. In this respect, it is noteworthy that vancomycin and the IRDye800CW have, as separate molecules, already been approved for clinical implementation. Importantly, as imaging is performed with an extremely low dose of vanco-800CW (0.07 nmol/mL), which is ~ 20- to 40-fold below the minimal inhibitory concentration (MIC) of vancomycin for staphylococci (https://eucast.org/clinical_breakpoints/), the possible side effects associated with vancomycin and a rapid selection for antimicrobial resistance are unlikely [11]. Consistent with this view, we observed thus far no effects of the tracer at these concentrations on culture results. We consider it therefore also unlikely that the described imaging approach could interfere with subsequent routine culturing. Furthermore, as vanco-800CW fluoresces in the NIR range, interfering tissue autofluorescence is minimal [18]. Indeed, in our human cadaver model, there was relatively little autofluorescence detectable. This has also been shown in live animal models [12] and oncologic TLFI studies where IRDye800CW was used [15].

Due to the characteristics of fluorophores and camera systems, TFLI is currently mainly suitable for imaging of superficial targets and intra-operative or endoscopic approaches. The NIR imaging system used in the present post-mortem experiment was, for example, designed for wide-field endoscopy and clinically implemented in the Gastroenterology and Hepatology departments of our hospital [19]. Our present study is the first in its kind that combines TFLI with arthroscopy. Importantly, by imaging the prosthesis intra-articular at the prosthetic surface, less fluorescence signal is lost compared with non-invasive imaging outside the knee joint. Accordingly, increased sensitivity and resolution in the detection of infection can be achieved [20]. This is underpinned by the observation that minor interruptions in the biofilm could arthroscopically be visualized with the fluorescence camera at submillimeter resolution (Fig. 6) during the post-mortem experiments.

In the clinic, around 60–70% of PJIs are caused by *S. aureus*, coagulase-negative staphylococci, streptococci, and enterococci, which are all GP bacteria, while less than 10% are caused by aerobic GN bacteria [8]. Judged by these percentages, vanco-800CW is suitable for the detection of PJIs in the majority of cases. This view is underscored by the finding that biomaterials coated with GP bacterial biofilms could, after treatment with vanco-800CW, all be easily discriminated from the tested GN bacteria and controls. The measured T/B ratios further showed that the GP bacterial strains with bound vanco-800CW emit fluorescence to a much higher level than the GN strains compared with the background. This finding is consistent with the notion that vanco-800CW is preferentially bound by GP bacteria [11]. The binding specificity of the fluorescently labelled vancomycin was further reinforced by the observed competitive inhibition of biofilm labelling through the pre-treatment of an *S. epidermidis* biofilm with non-labelled vancomycin at increasing concentrations. Observing that low amounts of vanco-800CW were also bound by the GN bacterial biofilms was surprising, but this finding can possibly be explained by the fact that peptidoglycan, the target for vanco-800CW, may not be fully covered in all instances by the GN bacterial outer membranes. This applies, for example, to dead bacteria. Whether this represents sufficient vanco-800CW accumulation to detect GN bacterial biofilms in a clinical context is yet to be explored. However, based on our findings, we anticipate that arthroscopic TFLI with vanco-800CW will have a much lower sensitivity for the detection of GN bacteria involved in PJI than for GP bacteria. Importantly, this potential limitation of the presently explored arthroscopic imaging approach can be overcome by multispectral arthroscopic imaging with multiple simultaneously applied tracers that target different microbial species, including GN bacteria. Considering that empirical antibiotic therapy may be less frequently needed, the direct diagnostic approach based on arthroscopic optical imaging is likely to result in better therapeutic regimens while, at the same time, less antimicrobial resistance may be elicited [21].

Some other limitations of this study need to be considered. Firstly, to reliably assess whether a prosthesis is infected, it also has to be scanned at spots were imaging is difficult. This mostly concerns the edges of the prosthesis and the prosthesis-bone interface. The imaging fiber used in the present experiment was not ideal for monitoring these spots due to a restricted maneuverability of the tip. This will also apply to the imaging of other joints, for example, hip prostheses, where arthroscopy is even more challenging. These practical restrictions still need to be addressed before clinical implementation can be considered. Secondly, careful selection of patients who might benefit from arthroscopic TFLI is important, as arthroscopy is a minimally invasive procedure and may also put patients at risk for new infections. Nonetheless, the risks of causing new infections though arthroscopy, mainly through the transfer of skin flora to the joint cavity, are estimated as very low (< 1%) [22]. Thirdly, the results of this study show that vanco-800CW is capable of detecting a representative panel of bacterial species in vitro. However, in the presented post-mortem arthroplasty experiments only a *S. epidermidis* strain was used. The reliability of this tool for the detection of other bacterial species is therefore yet to be established, and such experiments might include bacteria incapable of biofilm formation as negative controls. Finally, our in vitro method of biofilm formation on the prosthesis, in a laboratory setting with excess nutrients and controlled environmental conditions, does probably not fully resemble biofilms as they appear in vivo in patients. Nonetheless, we demonstrated the presence of EPS in the in vitro grown biofilms with Congo Red, which is proof of their authenticity [23]. However, biofilms grown in a laboratory setting with excess nutrients and controlled environmental conditions will be different from the ones that develop in patients. In addition, the arthroscopy experiments were conducted in a post-mortem setting, which undoubtedly presents differences with the actual in vivo situation. It will therefore be important to extend our proof-of-principle studies to in vivo arthroscopy in PJI animal models [24] and to examine whether vanco-800CW is capable of detecting real patient-derived biofilms (ex vivo) on infected biomaterials.

## Conclusion

This proof-of-principle study shows for a human prosthetic post-mortem infection model that arthroscopic detection and imaging of PJIs caused by GP bacteria is feasible with vanco-800CW. Its ability to directly and rapidly detect bacterial biofilms makes it stand out in comparison to other currently applied diagnostic approaches in clinical microbiology. This view is underpinned by the affinity of vanco-800CW for biofilms of a representative panel of clinically relevant bacterial species associated with biomaterial infections. We therefore believe that the here presented novel diagnostic modality holds great promise for clinical implementation with the exciting perspective of accurate and fast in situ diagnosis of PJIs.

## Electronic supplementary material

ESM 1 (DOCX 20 kb)

ESM 2 (MP4 46125 kb)

## Data Availability

All data in this manuscript will be freely available.
