# Supplementary material for: Image-guided in situ detection of bacterial biofilms in a human prosthetic knee infection model: a feasibility study for clinical diagnosis of prosthetic joint infections
Source: Eur J Nucl Med Mol Imaging. 2020 Sep 8;48(3):757–67. doi: 10.1007/s00259-020-04982-w (PMC8036220; doi:10.1007/s00259-020-04982-w)
Supplement: Supplementary file 1 — (DOCX 20 kb) [file 259_2020_4982_MOESM1_ESM.docx]

**Supplementary Materials**

**Image-guided *in situ* detection of bacterial biofilms in a human prosthetic knee infection model: A feasibility study for clinical diagnosis of prosthetic joint infections**

Jorrit W. A. Schoenmakers^1,2^, Marjolein Heuker^2*^, Marina López-Álvarez^2*^, Wouter B. Nagengast^3^, Gooitzen M. van Dam^4^, Jan Maarten van Dijl^2#^, Paul C. Jutte^1^, Marleen van Oosten^2^

^1^Department of Orthopaedics, University of Groningen, University Medical Center Groningen (UMCG), Groningen, The Netherlands

^2^Department of Medical Microbiology, University of Groningen, UMCG, Groningen, The Netherlands

^3^Department of Gastroenterology and Hepatology, University of Groningen, UMCG, Groningen, The Netherlands

^4^Department of Surgery, University of Groningen, UMCG, Groningen, The Netherlands

**^#^Corresponding author:** Prof. dr. Jan Maarten van Dijl, ORCID ID 0000-0002-5688-8438; Hanzeplein 1, PO Box 30001, 9700 RB Groningen, The Netherlands; email: [j.m.van.dijl01@umcg.nl](mailto:j.m.van.dijl01@umcg.nl), phone: +31503615187

***Equal contributions**

**Running title**: Optical arthroscopy to detect infection

**Supplementary Table 1.**

| **Bacterial strain** | **Average fluorescence source intensity per ROI per species** | **Average fluorescence background intensity per ROI per species** | **Average T/B ratio per species** | **Average colony forming units per species** |
| --- | --- | --- | --- | --- |
| *S. aureus* | 4429 | 254.5 | 18.42 | 6.61 x 10^8^ |
| *S. caprae* | 9733.5 | 254.5 | 36.28 | 2.22 x 10^10^ |
| *S. epidermidis* | 3962 | 276 | 14.15 | 1.70 x 10^9^ |
| *S. lugdunensis* | 9510.5 | 273 | 33.69 | 1.99 x 10^9^ |
| *E. faecalis* | 7767.5 | 255.5 | 29.97 | 5.03 x 10^9^ |
| *S. epidermidis* ATCC 35984 | 15759.5 | 219.5 | 56.5 | 1.75 x 10^7^ |
| *E. coli* | 962.5 | 267.5 | 3.48 | 3.24 x 10^8^ |
| *P. aeruginosa* | 859 | 267.5 | 3.15 | 3.52 x 10^8^ |
| *Sterile* | 220.5 | 261 | 0.86 | 0 |

**Supplementary Table 1. Average fluorescence source and background intensities, calculated averaged target-to-background (T/B) ratios per bacterial strain and colony forming units.**

14 bacterial strains were collected and used to grow *in vitro* biofilms on cobalt-chrome discs. These included *S*. *aureus* (2 isolates; Gram-positive (GP)), *S. caprae* (2 isolates, GP), *S. epidermidis* (2 isolates, GP), *S. lugdunensis* (2 isolates, GP), *E. faecalis* (2 isolates, GP), *E. coli* (2 isolates; Gram-negative (GN)), and *P. aeruginosa* (2 isolates, GN). In addition, the *S. epidermidis* ATCC 35984 strain was included for control, as well as 2 sterile cobalt-chrome discs. All discs were treated with vanco-800CW. Fluorescence images were recorded using the SurgVision Explorer Air (Settings for fluorescence measurements: fluorescence exposure 25 ms, fluorescence gain 300; SurgVision, the Netherlands). Data was analyzed using ImageJ (National Institutes of Health, Maryland, US). Regions of interest (ROIs) were drawn around the biomaterials after which the fluorescence signal was quantified. The background signal was quantified by drawing a ROI off-target in the background of the same image. To determine target-to-background (T/B) ratios, ROIs were divided by the background fluorescence. The discs were sonicated after which serial dilutions of the sonication-fluid were made. Samples were plated on blood agar plates (5% sheep blood) and average numbers of colony forming units (CFUs) were determined. It should be noted that CFU counting after sonication is a semi-quantitative method, and that the yield after sonication can vary per bacterial species. Therefore, it is not possible to directly link a fluorescence signal to the respective CFU count. Nonetheless, the present data imply that comparable biofilms of GP and GN bacterial species were formed in the present experiments, whereas only the GP biofilms were detected with vanco-800CW.

***Movie 1. Targeted optical arthroscopy to detect bacterial biofilm***The supplemental movie 1 provides a theoretical and practical overview of the image-guided *in situ* detection of bacterial biofilms in a human prosthetic knee infection model.
